# Identification of Immune Cell Infiltration in Murine Pheochromocytoma during Combined Mannan-BAM, TLR Ligand, and Anti-CD40 Antibody-Based Immunotherapy

**DOI:** 10.3390/cancers13163942

**Published:** 2021-08-05

**Authors:** Ondrej Uher, Thanh-Truc Huynh, Boqun Zhu, Lucas A. Horn, Veronika Caisova, Katerina Hadrava Vanova, Rogelio Medina, Herui Wang, Claudia Palena, Jindrich Chmelar, Zhengping Zhuang, Jan Zenka, Karel Pacak

**Affiliations:** 1 Section on Medical Neuroendocrinology, *Eunice Kennedy Shriver* National Institute of Child Health and Human Development, NIH, Bethesda, MD 20892, USA; ondrej.uher@nih.gov (O.U.); huynht@mail.nih.gov (T.-T.H.); zhu.boqun@zs-hospital.sh.cn (B.Z.); vcaisova@childrensnational.org (V.C.); katerina.hadravavanova@nih.gov (K.H.V.); 2 Department of Medical Biology, Faculty of Science, University of South Bohemia, 37005 Ceske Budejovice, Czech Republic; chmelar@prf.jcu.cz (J.C.); jzenka@prf.jcu.cz (J.Z.); 3 Endoscopy Center and Endoscopy Research Institute, Zhongshan Hospital, Fudan University, Shanghai 200032, China; 4 Laboratory of Tumor Immunology and Biology, Center for Cancer Research, National Cancer Institute, NIH, Bethesda, MD 20892, USA; lucas.horn@nih.gov (L.A.H.); palenac@mail.nih.gov (C.P.); 5 Neuro-Oncology Branch, National Cancer Institute, NIH, Bethesda, MD 20892, USA; rogelio.medina@nih.gov (R.M.); herui.wang@nih.gov (H.W.); zhengping.zhuang@nih.gov (Z.Z.)

**Keywords:** pheochromocytoma, intratumoral immunotherapy, immune memory, toll-like receptor, bilateral tumor model

## Abstract

**Simple Summary:**

Multiple types of primary tumors and metastases that present with very little if any immune cell infiltration (so-called immunologically “cold” tumors) do not respond to current immunotherapies. In this study, we show that recently developed intratumoral application-based immunotherapy using mannan-BAM, TLR ligands, and anti-CD40 antibody (MBTA therapy) efficiently suppresses tumor growth in a murine bilateral pheochromocytoma model. Moreover, MBTA therapy increases the recruitment of innate immune cells followed by adaptive immune cells not only to primary (injected) tumors but also distal (non-injected) tumors. We also demonstrated that after successful MBTA therapy of subcutaneous pheochromocytoma, long-term immunological memory is driven by CD4^+^ T cells. Taken together, this study helps to better understand the systemic effect of MBTA therapy and its use for tumor and metastasis reduction or even elimination.

**Abstract:**

Immunotherapy has become an essential component in cancer treatment. However, the majority of solid metastatic cancers, such as pheochromocytoma, are resistant to this approach. Therefore, understanding immune cell composition in primary and distant metastatic tumors is important for therapeutic intervention and diagnostics. Combined mannan-BAM, TLR ligand, and anti-CD40 antibody-based intratumoral immunotherapy (MBTA therapy) previously resulted in the complete eradication of murine subcutaneous pheochromocytoma and demonstrated a systemic antitumor immune response in a metastatic model. Here, we further evaluated this systemic effect using a bilateral pheochromocytoma model, performing MBTA therapy through injection into the primary tumor and using distant (non-injected) tumors to monitor size changes and detailed immune cell infiltration. MBTA therapy suppressed the growth of not only injected but also distal tumors and prolonged MBTA-treated mice survival. Our flow cytometry analysis showed that MBTA therapy led to increased recruitment of innate and adaptive immune cells in both tumors and the spleen. Moreover, adoptive CD4^+^ T cell transfer from successfully MBTA-treated mice (i.e., subcutaneous pheochromocytoma) demonstrates the importance of these cells in long-term immunological memory. In summary, this study unravels further details on the systemic effect of MBTA therapy and its use for tumor and metastasis reduction or even elimination.

## 1. Introduction

The currently increased interest in cancer immunotherapy is based on numerous clinical benefits in the treatment of different tumor types [1]. Nevertheless, systemic delivery of immunotherapeutic drugs, especially various checkpoint inhibitors, might cause severe off-target toxicities, reportedly exhibiting a dose-related effect [2]. Moreover, these therapies are quite expensive, and in some tumors, the therapeutic outcomes are suboptimal. In contrast, intratumoral therapies result in high drug concentration within the tumor microenvironment, while small amounts of drugs are used, leading to lower systemic drug concentration, reduced side effects, and substantial cost reduction [3]. Furthermore, several recent studies have shown that intratumoral drug administration could induce a systemic immune response even in distal non-treated tumors, followed by their successful growth inhibition or even elimination [3,4].

Pheochromocytoma (PHEO) and paraganglioma (PGL) are rare neuroendocrine tumors derived from neural crest cells [5]. Up to 30% of all PHEO/PGL cases develop metastases for which therapeutic options are still very limited [6]. PHEO/PGL can also be considered as immunologically “cold” tumors due to their low amounts of neoantigens, low somatic mutation burden, and lack of leukocyte infiltration [7,8,9]. Currently, there are only two clinical trials that include immunotherapy in metastatic/inoperable PHEO/PGL, specifically, systemic application of different checkpoint inhibitors nivolumab + ipilimumab and pembrolizumab (identifiers: NCT02834013 and NCT02721732) [10].

The basics of the immunotherapeutic approach used here were published previously by our group and are based on the intratumoral injection of mannan anchored to the tumor cell surface via a biocompatible anchor for cell membrane (BAM), a mixture of toll-like receptor (TLR) ligands, and agonistic anti-CD40 monoclonal antibody (MBTA therapy). Mannan is a *Saccharomyces cerevisiae*-derived polysaccharide that serves as a ligand stimulating phagocytosis via the activation of the complement lectin pathway and resulting in iC3b production and subsequent tumor cell opsonization [11,12,13]. TLR ligands support the infiltration of immune cells into tumors, as well as their activation. The combination of TLR ligands (resiquimod, polyinosinic-polycytidylic acid, and lipoteichoic acid) has been previously assessed as the most potent approach. Resiquimod (R-848) is an imidazoquinolinamine and synthetic analog of viral ssRNA that activates immune cells via the TLR7/8 and TLR7 pathways in humans and mice, respectively [14]. Polyinosinic-polycytidylic acid (poly(I:C)) is a synthetic analog of dsRNA that activates immune cells via the TLR3 pathway [15]. Finally, lipoteichoic acid (LTA) is a constituent of the *Bacillus subtilis* cell wall, activating the immune cells via the TLR2 pathway [16]. Anti-CD40 is an agonistic antibody that mimics the CD40 ligand expressed on helper (CD4^+^) T cells. The ligation of CD40 on macrophages, dendritic cells, and B cells leads to activation of these cells, enhances antigen presentation, and induces an effective T cell anti-tumor response [17,18,19]. Previous research publications, leading to the current components of MBTA therapy, have been published here [12,20,21] and summarized in this review [22].

MBTA therapy has been previously tested in various subcutaneous murine models. In the melanoma B16-F10 model, MBTA therapy, even without anti-CD40, demonstrated the eradication of tumors in 83% of treated animals. In the aggressive pancreatic adenocarcinoma model (Panc02), the efficacy of therapy was observed in 80% of the treated animals [20]. When tested in primary and bilateral tumor models of colon carcinoma (CT26), MBTA therapy resulted in the slower progression and prolonged survival of the treated animals [23]. Finally, in the murine subcutaneous PHEO model, MBTA therapy resulted in the complete elimination of tumors in 62.5% of mice, significantly decreased the bioluminescence signal intensity of metastatic organ lesions, and prolonged the survival of MBTA-treated mice when tested on metastatic PHEO [24].

All the aforementioned studies have focused heavily on local immune responses in MBTA-injected tumors. Therefore, in the present study, we assessed immune responses induced by intratumoral MBTA therapy using a bilateral PHEO tumor model that mimics the presence of both primary and distal tumors and enables the real-time analysis of both tumor types, including the assessment of immune cell trafficking into these tumors [25,26]. First, we established a bilateral PHEO model and studied the systemic immune response during intratumoral MBTA therapy in primary (injected) and distal (non-injected) tumors. We demonstrate that MBTA therapy could suppress the growth of not only injected but also distal tumors. Second, we describe the detailed infiltration of innate and adaptive cells in injected and distal tumors, as well as the changes in the spleen during MBTA therapy. Flow cytometry analysis showed the ability of MBTA therapy to increase the recruitment of innate and adaptive immune cells in both tumors and the spleen. Finally, we demonstrated the importance of CD4^+^ cells in immunological memory after successful MBTA therapy. The results of this study are essential for a better understanding of how MBTA therapy contributes to tumor growth reduction or even elimination, including metastases. Such an understanding would further help optimize this therapeutic approach with future vision using it in clinical trials to fight metastatic cancers.

## 2. Materials and Methods

### 2.1. Mannan-BAM Synthesis and MBTA Therapy

Mannan from *Saccharomyces cerevisiae*; lipoteichoic acid (LTA) from *Bacillus subtilis*; polyinosinic-polycytidylic acid, sodium salt (poly(I:C)); ammonium acetate, and natrium cyanoborohydride were obtained from Sigma-Aldrich (St. Louis, MO, USA). BAM was obtained from NOF Corporation (White Plains, NY, USA). Resiquimod (R-848) was obtained from Tocris Bioscience (Minneapolis, MN, USA). Monoclonal anti-CD40 (clone FGK4.5/FGK45) was obtained from BioXCell (West Lebanon, NH, USA). Aminated mannan was prepared by reductive amination. Mannan solution in an environment of ammonium acetate (300 mg/mL) was reduced by 0.2 M natrium cyanoborohydride at pH 7.5 and 50 °C for five days. The solution was further dialyzed using a MWCO 3500 dialysis tube (Serva, Heildelberg, Germany) against PBS at 4 °C overnight. Binding of BAM on the amino group of mannan was performed at pH 7.3 according to the methodology described by Kato et al. [27]. For 1 h at room temperature, the N-hydroxysuccinimide (NHS) group of BAM was allowed to react with the amino group of mannan. Solution of mannan-BAM was obtained after dialysis, as described above, and stored frozen at −20 °C. Mice were treated intratumorally on days 0, 1, 2, 8, 9, 10, 16, 17, 18, 24, 25, and 26 with 50 μL of MBTA therapeutic mixture consisting of 0.5 mg R-848 (HCl form), 0.5 mg poly(I:C), 0.5 mg LTA, and 0.4 mg anti-CD40 per mL of 0.2 mM mannan-BAM in PBS. MBTA therapy was always only applied to the right tumor.

### 2.2. Cell Lines, Mice, and Tumor Establishment

The MTT-luc cells (mouse tumor tissue–luciferase cells) used in this study are rapidly growing cells derived from liver metastases of MPC (mouse pheochromocytoma cells) and transfected with a luciferase plasmid [28,29]. The cells were maintained in Dulbecco’s modified Eagle medium (DMEM) (Sigma-Aldrich) supplemented with 10% of heat-inactivated fetal bovine serum (Gemini, West Sacramento, CA, USA), 100 U/mL of penicillin/streptomycin (Gemini), and 750 μg/mL of geneticin (Thermo Fisher Scientific, Waltham, MA, USA) for stable cell line selection. Cells were cultured at 37 °C in humidified air with 5% CO_2_. The cell lines were tested for mycoplasma using a MycoAlert™ detection kit purchased from Lonza (Walkersville, MD, USA).

Female B6(Cg)- *Tyr^c−2 J^*/J (B6 albino) mice were purchased from the Jackson Laboratory (Bar Harbor, ME, USA). Mice were housed in specific pathogen-free barrier facilities with free access to sterile food and water, with a photoperiod of 12/12.

For the subcutaneous PHEO model, mice were subcutaneously injected in the previously shaved right flank with 3 × 10^6^ MTT-luc cells in DMEM (0.2 mL) without additives. For the bilateral PHEO model, mice were subcutaneously injected in both the previously shaved right and left flank with 3 × 10^6^ MTT-luc cells in 0.2 mL of DMEM without additives (each site).

### 2.3. Antibodies for Flow Cytometry

Antibodies purchased from BioLegend (San Diego, CA, USA) included PerCP-Cy5.5 anti-mouse/human CD44 (clone IM7), Alexa Fluor^®^ 700 anti-mouse CD3ε (clone 500A2), APC/Cy7 anti-mouse CD45 (clone 30-F11), Brilliant Violet 785^TM^ anti-mouse CD8a (clone 53-6.7), Brilliant Violet 711^TM^ anti-mouse CD4 (clone RM4-5), Brilliant Violet 785^TM^ anti-mouse CD19 (clone 6D5), APC anti-mouse/human CD11b (clone M1/70), PeCP-Cy5.5 anti-mouse Ly-6C (clone HK1.4), FITC anti-mouse Ly-6G (clone 1A8), Brilliant Violet 711^TM^ anti-mouse F4/80 (clone BM8), Alexa Fluor^®^ 700 anti-mouse I-A/I-E (clone M5/114.15.2), Brilliant Violet 605^TM^ anti-mouse CD335 (NKp46) (clone 29A1.4), Brilliant Violet 421^TM^ anti-mouse CD62L (clone MEL-14), APC anti-mouse CD366 (Tim-3) (clone B8.2C12), PE anti-mouse CD279 (PD-1) (clone 29F.1A12), and Brilliant Violet 421^TM^ anti-mouse CD38 (clone 90). BV605 anti-mouse CD11c (clone HL3) was purchased from BD Biosciences (San Jose, CA, USA).

### 2.4. Isolation of Cells Infiltrating the Tumors and Spleen and Flow Cytometry Analysis

Tumor-bearing mice were euthanized by cervical dislocation, followed by the collection of blood samples, spleens, and tumors. Spleens were processed into single-cell suspensions via mechanical dissociation, filtration (70 μm), and erythrocyte lysis. Tumors were weighed, mechanically dissociated, and digested using a digestion cocktail composed of collagenase types I and IV (both 1 mg/mL) (Thermo Fisher Scientific) and deoxyribonuclease I (40 U/mL) (Invitrogen, Carlsbad, CA, USA). After an incubation of 1 h at 37 °C with constant agitation, the samples were centrifuged and the supernatants were collected and used for ELISA-based cytokine detection. Cell pellets were passed through 70 μm filters, then tumor-infiltrating cells were isolated by density gradient centrifugation. Briefly, each pellet was resuspended in 40% Percoll^TM^ PLUS (GE Healthcare, Chicago, IL, USA)/Hanks’ balanced salt solution, overlaid with a 70% Percoll^TM^ PLUS/HBSS, and the interface was isolated after 30 min of centrifugation (800× *g*). Subsequently, the cells were incubated for 30–45 min with the antibodies. Dead cells were excluded using the LIVE/DEAD^TM^ Fixable Aqua Dead Cell Stain Kit (Thermo Fisher Scientific). CountBright^TM^ absolute counting beads (Invitrogen) were used to count the absolute numbers of individual CD45^+^ cells. All analyses were performed using an Attune NxT Flow Cytometer (Thermo Fisher Scientific) and interpreted using the FlowJo^TM^ software (version 10.6.1, Beckon Dickinson, Ashland, OR, USA). The flow cytometry analysis of immune cell subsets was defined as follows: CD4^+^ T cells = CD45^+^ CD3^+^ CD4^+^; CD8^+^ T cells = CD45^+^ CD3^+^ CD8^+^; TCM = CD45^+^ CD3^+^ CD44^+^ CD62L^+^; TE/EM = CD45^+^ CD3^+^ CD44^+^ CD62L^−^; neutrophil = CD45^+^ CD11b^+^ F4/80^−^ Ly6G^+^ Ly6C^lo^; monocyte = CD45^+^ CD11b^+^ F4/80^−^ Ly6G^-^ Ly6C^hi^; dendritic cell = CD45^+^ CD11b^−^ F4/80^−^ CD11c^+^; NK cell = CD45^+^ CD3^+^ CD335^+^; macrophage = CD45^+^ CD11b^+^ F4/80^+^, M1-like macrophage = CD45^+^ CD11b^+^ F4/80^+^ CD279^−^ CD38^hi^; M2-like macrophage = CD45^+^ CD11b^+^ F4/80^+^ CD279^+/−^ CD38^lo^. A representative gating strategy could be found in the Supplementary Materials (Figures S2 and S3). The M1/M2 ratios were calculated as percentages of M1-like macrophages divided by M2-like macrophages.

### 2.5. Adoptive CD4^+^ and CD8^+^ T Cell Transfer

Splenocytes were isolated from cured MBTA-treated donor mice previously bearing subcutaneous PHEO (MTT-luc cells) and untouched control donor mice of the same age (*n* = 5 per group). CD4^+^ or CD8^+^ T cells were isolated from a single-cell suspension of splenocytes after ACK lysis using a MACS negative selection kit (Miltenyi Biotec, Auburn, CA, USA). The procedures were performed according to the manufacturer’s instructions. Subsequently, cells were diluted in PBS and 2.5 × 10^6^ cells (CD4^+^ or CD8^+^ T cells) in a volume of 0.2 mL were intraperitoneally injected into recipient mice (*n* = 5 per group). The purity of the preparations was 94 and 93% for the CD4^+^ and CD8^+^ T cells, respectively. After 7 days, recipient mice received a subcutaneous injection of MTT-luc cells as described above.

### 2.6. Cytokine Assay

Tumor supernatants and plasma collected during the analysis of tumor-infiltrating cells were used to measure IFN-γ and IL-10 levels, using the IFN-γ ELISA Kit, Extra Sensitive (Thermo Fisher Scientific, Waltham, MA, USA), and IL-10 (LSBio, Seattle, WA, USA) for detection.

### 2.7. Data and Statistical Analysis

The tumor volume was measured with a caliper and calculated as $V=\left(\pi /6\right)AB^{2}$ (where A and B stand for the largest and the smallest dimensions of the tumor, respectively). For the tumor growth analysis, the area under the curve (AUC) was calculated until treatment day 20. Statistical analysis was performed on these AUC values using *t*-test (Mann–Whitney test). Reduction of tumor growth (%) was determined as follows: $\frac{\left(AUC\;value\;in\;control\;group-AUC\;value\;in\;treated\;group\right)\times 100}{AUC\;value\;in\;control\;group}.$ Kaplan–Meier survival curves were compared using a log-rank test. For the flow cytometry and ELISA test data analysis, multiple *t*-tests were used. Data were analyzed using the GraphPad Prism software version 8 for Mac OS (GraphPad Software, La Jolla, CA, USA). The error bars indicate the standard errors of the mean (SEM). The pictures were created using BioRender.com.

## 3. Results

### 3.1. Effect of MBTA Therapy on the Bilateral PHEO Model

**Figure 1:**
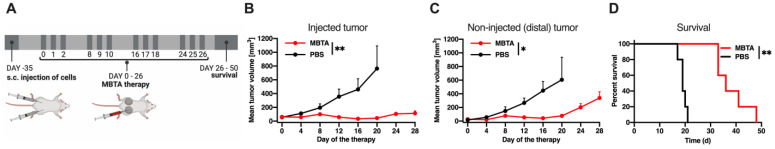
Bilateral subcutaneous PHEO mouse model. (**A**) Experimental design diagram of MBTA therapy in the bilateral subcutaneous PHEO mouse model. (**B**) The tumor volume growth of the injected and (**C**) the distal tumors presented as growth curves (*n* = 5 per group) (* *p* < 0.05, ** *p* < 0.01). (**D**) The survival analysis presented as a Kaplan–Meier curve (** *p* < 0.01).

To better understand the systemic immune response generated by the intratumoral MBTA therapy and immune cell trafficking into injected (primary) and non-injected (distal) tumors, we first established a bilateral PHEO model. Mice were subcutaneously injected with MTT-luc cells in both the right and left flanks. After 35 days, the average tumor volume on the right and left sites were 59.5 and 22.0 mm^3^, ranging between 6.9–118.0 and 6.0–34.9 mm^3^. MBTA therapy was applied to the primary tumor, and both the primary and distal tumors were observed for size changes (Figure 1A). The growth of the MBTA-injected (Figure 1B) and distal tumors (Figure 1C) got reduced during MBTA therapy, although it was not completely eliminated. Until day 20 from the start of the treatment, the reduction of tumor growth of the MBTA-injected tumors and the distal tumors was 76.8% and 72.7%, respectively. However, both the MBTA-injected and distal tumors started to grow after day 20, despite ongoing MBTA therapy (last treatment cycle was given at days 24–26). Non-injected (distal) tumors showed a strong increase in tumor volume, whereas MBTA-injected tumors showed only a moderate increase. Nevertheless, we observed the prolonged survival of MBTA-treated mice compared to the control (Figure 1D). These results confirmed that the intratumoral application of MBTA therapy in primary tumors induced systemic effects on distal tumors, resulting in significantly improved survival of the MBTA-treated mice.

### 3.2. Characterization of Tumor Infiltrating and Splenic Leukocytes, as Well as Innate Immune Cells during MBTA Therapy

**Figure 2:**
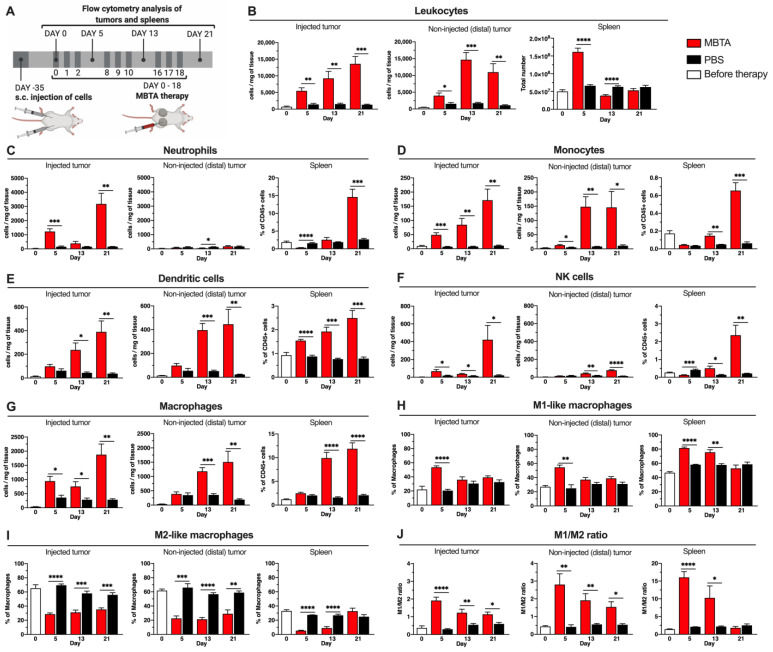
Flow cytometry analysis of tumor infiltrating leukocytes and innate immune cells. (**A**) Experimental design diagram of the flow cytometry analysis. The results from the analysis of injected and distal tumors and spleens: (**B**) leukocytes (CD45^+^), (**C**) neutrophils (CD45^+^ CD11b^+^ F4/80^−^ Ly6G^+^ Ly6C^lo^), (**D**) monocytes (CD45^+^ CD11b^+^ F4/80^−^ Ly6G^−^ Ly6C^hi^), (**E**) DCs (CD45^+^ CD11b^−^ F4/80^−^CD11c^+^), (**F**) NK cells (CD45^+^ CD3^+^ CD335^+^), (**G**) Macrophages (CD45^+^ CD11b^+^ F4/80^+^), (**H**) M1-like macrophages (CD45^+^ CD11b^+^ F4/80^+^ CD279^−^ CD38^hi^), (**I**) M2-like macrophages (CD45^+^ CD11b^+^ F4/80^+^ CD279^+/−^ CD38^lo^), (**J**) M1/M2 ratio (*n* = 5 per group/day) (* *p* < 0.05, ** *p* < 0.01, *** *p* < 0.001, **** *p* < 0.0001).

First, flow cytometry analyses of tumor-infiltrating leukocytes were performed to assess the detailed immune profiles of injected (primary) and non-injected (distal) tumors, as well as those of spleens during MBTA or PBS therapy (Figure 2A). The results from these analyses revealed that intratumoral MBTA therapy significantly increased the number of leukocytes (CD45^+^ cells) in both the injected and distal tumors compared to the control (Figure 2B). The number of splenic leukocytes significantly increased only on day 5 after the first MBTA injection cycle (Figure 2B). Further assessment of innate leukocyte subpopulations revealed striking and site-specific inflammatory responses in the MBTA-treated mice. Neutrophils were significantly increased in the injected but almost absent in the distal tumors during MBTA therapy, while in the spleen, neutrophils were elevated after the third therapeutic cycle (day 21) (Figure 2C). The number of monocytes was elevated in both tumor types of MBTA-treated mice during the entire therapy and increased in the spleen from day 13 (Figure 2D). The number of dendritic cells (DCs) was significantly elevated in the injected and distal tumors after the second MBTA therapeutic cycle (day 13). Interestingly, the number of DCs in the spleen consistently increased during the therapy (Figure 2E). The number of natural killer (NK) cells in the MBTA-injected tumors were significantly increased 5 days after the start of the treatment, while distal tumors demonstrated such an increase 13 days after the start of the treatment (Figure 2F).

We also focused on two major phenotypes of macrophages, M1-like and M2-like macrophages. The overall macrophage number significantly increased directly after the first MBTA therapeutic cycle (day 5) in the injected tumors. Such an increase was observed in distal tumors and in the spleen after the second therapeutic cycle (day 13) (Figure 2G). In both injected and distal tumors, the percentage of classically activated M1-like macrophages significantly increased only on day 5 (Figure 2H). Of note, the percentage of alternatively activated M2-like macrophages significantly decreased in injected and distal tumors as well as in the spleens of MBTA-treated mice when compared to the control group throughout the treatment course (Figure 2I). Thus, the ratio of M1-like to M2-like macrophages (M1/M2) in both tumors and spleens was significantly higher in the MBTA-treated group than in the control group (Figure 2J), suggesting the ability of MBTA therapy to systematically activate macrophages, increase trafficking into tumors, and decrease the induction of immunosuppressive M2-like phenotype.

When MBTA-injected tumors were compared with distal tumors, all types of innate cells, except DCs, were significantly elevated only in MBTA-injected tumors and not in distal tumors after the first cycle of treatment (day 5), underscoring MBTA’s ability to induce an immune response first in injected tumors and subsequently in distal tumors (Figure S1).

In summary, these results demonstrate that intratumoral injection of MBTA therapy significantly augmented the trafficking of innate immune cells not only within distal non-treated tumors and within the spleen.

### 3.3. Characterization of Tumor Infiltrating and Splenic Lymphocytes during MBTA Therapy

**Figure 3:**
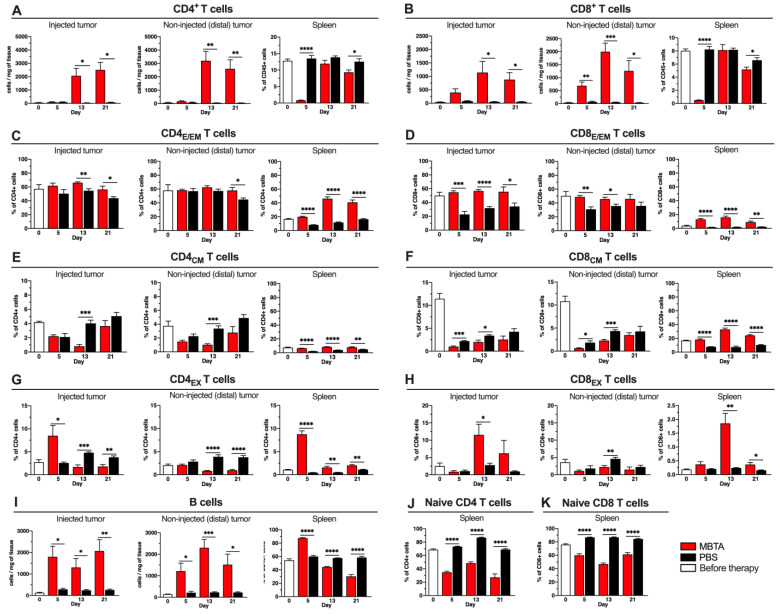
Flow cytometry analysis of tumor infiltrating lymphocytes and their subpopulations in injected and distal tumors. B6(Cg)- *Tyr^c−2J/^*J mice were treated and flow cytometry analysis was performed as described in Figure 2. (**A**) CD4^+^ T cells (CD45^+^ CD3^+^ CD4^+^), (**B**) CD8^+^ T cells (CD45^+^ CD3^+^ CD8^+^), (**C**) CD4_E/EM_ T cells (CD45^+^ CD3^+^ CD4^+^ CD44^+^ CD62L^−^), (**D**) CD8_E/EM_ T cells (CD45^+^ CD3^+^ CD8^+^ CD44^+^ CD62L^−^), (**E**) CD4_CM_ T cells (CD45^+^ CD3^+^ CD4^+^ CD44^+^ CD62L^+^), (**F**) CD8_CM_ T cells (CD45^+^ CD3^+^ CD8^+^ CD44^+^ CD62L^+^), (**G**) CD4_EX_ T cells (CD45^+^ CD3^+^ CD4^+^ PD1^+^ TIM3^+^), (**H**) CD8_EX_ T cells (CD45^+^ CD3^+^ CD8^+^ PD1^+^ TIM3^+^), (**I**) B cells (CD45^+^ CD19^+^), (**J**) Naïve CD4 T cells (CD45^+^ CD3^+^ CD4^+^ CD44^−^ CD62L^+^), (**K**) Naïve CD8 T cells (CD45^+^ CD3^+^ CD8^+^ CD44^−^ CD62L^+^) (*n* = 5 per group/day) (* *p* < 0.05, ** *p* < 0.01, *** *p* < 0.001, **** *p* < 0.0001).

Tumor-infiltrating lymphocytes (TILs) and their subpopulations were similarly explored using flow cytometry. TIL analyses showed strong infiltration of helper CD4^+^ and cytotoxic CD8^+^ T cells in both the injected and distal tumors (Figure 3A,B). The CD4^+^ T cells significantly increased after the second MBTA therapeutic cycle (day 13). In contrast, in both tumors, CD8^+^ T cells were already elevated after the first therapeutic cycle but statistical significance could be observed only in distal tumors. The analysis of the splenic T cells revealed a strong decrease in CD4^+^ and CD8^+^ T cells directly after the first therapeutic cycle compared to the control. A higher increase in the proportion of effector/effector memory T cells (E/EM, CD44^+^ CD62L^−^) in the tumors of MBTA-treated mice during the entire therapy was observed in CD8_E/EM_ and less so in CD4_E/EM_. The proportion of splenic CD4_E/EM_ and CD8_E/EM_ T cells was significantly elevated during the entire MBTA therapy compared to that in the control (Figure 3C,D). The assessment of the central memory (CM, CD44^+^ CD62L^+^) CD4^+^ or CD8^+^ T cells in the tumors did not show a significantly higher percentage of these cells in the MBTA-treated group. In contrast, a significant increase in the percentage of both CD4_CM_ and CD8_CM_ T cells could be observed in the spleens of MBTA-treated mice compared to the control (Figure 3E,F). We could observe differences in the exhausted T cells (EX, PD1^+^ TIM3^+^) in the tumors in CD4_EX_, where MBTA-injected tumors displayed a higher proportion of these cells on day 5 compared to the control. However, at the end of the analysis, a significantly higher CD4_EX_ T cell percentage proportion could be observed in the tumors of the control group than in those of the MBTA-treated group. The splenic CD4_EX_ T cells in the MBTA-treated mice were significantly higher during the entire therapy than in the control. We could observe significant increases in the CD8_EX_ T cells of the MBTA-treated mice only in the injected tumor and spleen on day 13 (Figure 3G,H).

Splenic naïve CD4^+^ and CD8^+^ T cells were significantly lower in the MBTA-treated mice than in the control during the entire therapy (Figure 3J,K). The B cell analysis showed strong infiltration in both tumors of the MBTA-treated mice. Interestingly, the number of splenic B cells was highest after the first therapeutic, and 90% of all leukocytes in the spleen were B cells. However, after day 5, the number of B cells in the spleen constantly decreased (Figure 3I).

In summary, CD4^+^ T cells were the most abundant T lymphocytes in both the injected and distal tumors. The highest changes in the TIL subpopulation percentage proportions were observed in the CD8_E/EM_ T cells in both the injected and distal tumors of the MBTA-treated mice. All T lymphocyte splenic subpopulations, except for the naïve T cells, were elevated during MBTA therapy compared to those in the control, suggesting strong T cell activation upon MBTA therapy.

### 3.4. IFN-γ and IL-10 Level Detection in the Bilateral PHEO Model

**Figure 4:**
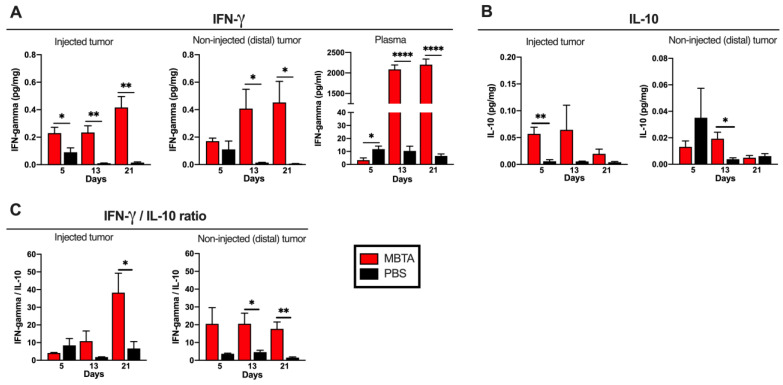
IFN-γ and IL-10 levels in the injected and distal tumors. (**A**) IFN-γ levels and (**B**) IL-10 levels from tumor samples measured by ELISA. (**C**) IFN-γ/IL-10 ratio in tumors (*n* = 5 per group/day) (* *p* < 0.05, ** *p* < 0.01, **** *p* < 0.0001).

To characterize whether the immune response in the injected and distal tumors is pro- or anti-inflammatory, we measured the IFN-γ and IL-10 levels. The ELISA assays revealed significantly elevated pro-inflammatory IFN-γ levels in both tumors, mostly after the second MBTA therapeutic cycle on day 13 (Figure 4A). The level of anti-inflammatory IL-10 in the MTBA tumors were 8-fold lower (~0.05 pg/mg) than the level of IFN-γ (~0.4 pg/mg). More precisely, a significant IL-10 level difference could be observed in MBTA-injected tumors on day 5 and in distal tumors on day 13 (Figure 4B). The IFN-γ plasma levels in MBTA-treated mice were 200-fold higher (~2000 pg/mL) than in the control (~10 pg/mL), whereas the IL-10 plasma levels were below the detection limit. When we calculated the IFN-γ/IL-10 ratio, we detected significant differences in the injected tumors on day 21 and in distal tumors on days 13 and 21, underscoring the pro-inflammatory microenvironment that was established by the MBTA therapy (Figure 4C).

### 3.5. The Immune Memory after MBTA Therapy Is CD4^+^ T Cell-Dependent

**Figure 5:**
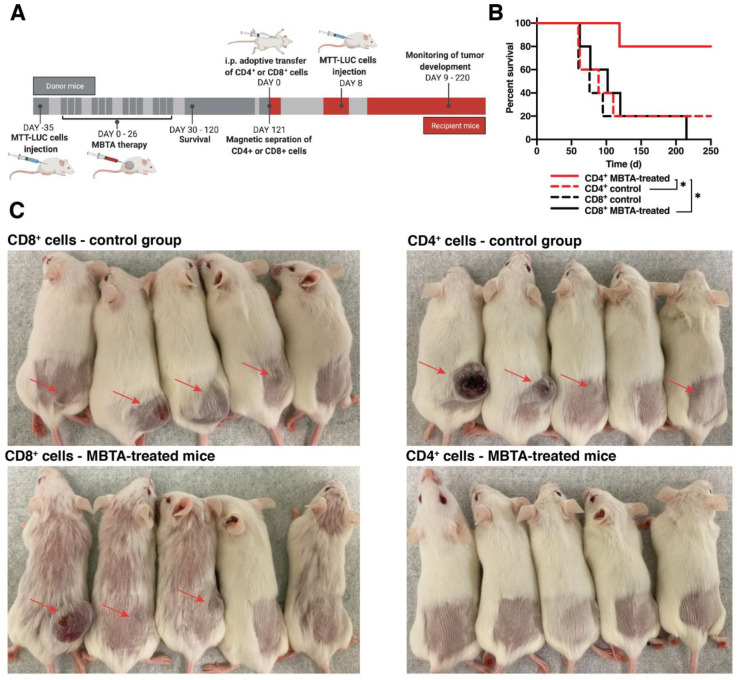
Adoptive transfer of CD4^+^ or CD8^+^ T cells. (**A**) Experimental design diagram of adoptive transfer. (**B**) The survival analysis is presented as a Kaplan-Meier curve (*n* = 5 per group) (* *p* < 0.05). (**C**) Pictures of recipient mice taken after 60 days from the MTT-luc inoculation.

To determine whether immunological memory against PHEO following MBTA therapy could be generated by CD4^+^ or CD8^+^ T cells, we performed their adoptive transfer. Mice were injected with MTT-luc cells in the right flank (subcutaneous PHEO), and after tumor development, they were treated with MBTA therapeutic mixture as described above. Mice that remained tumor-free for 120 days after the start of MBTA therapy were used as donor mice. Seven days after adoptive transfer of CD4^+^ or CD8^+^ T cells, recipient mice were subcutaneously injected with MTT-luc cells in the right flank and were monitored for PHEO tumor development (Figure 5A).

Interestingly, flow cytometry analysis, performed 120 days after the start of treatment, revealed no significant changes in the numbers of leukocytes, CD4^+^, and CD8^+^ T cells, and their effector/effector memory, central memory, and exhausted subpopulations in spleens of MBTA-treated mice when compared to the same age control mice.

On day 60 after the adoptive transfer, tumor rejection was observed in 40% of recipient mice with CD8^+^ T cells from MBTA-treated mice (2 from 6 mice), 100% of recipient mice with CD4^+^ T cells from MBTA-treated mice, and in 20% of both control groups with CD4^+^ or CD8^+^ T cells (Figure 5C). Mice were observed for tumor development for 250 days after adoptive transfer and by the end of this observation period, all mice that received donor CD8^+^ T cells from MBTA treated mice developed tumor, whereas 80% of mice that received donor CD4^+^ T cells from MBTA treated mice were protected against the tumor development (Figure 5B). Collectively, these data suggest the importance of CD4^+^ T cells in the rejection of tumor cells after successful MBTA therapy.

## 4. Discussion

In the present study, we focused on MBTA therapy-induced systemic immune responses. First, we used a bilateral PHEO model, in which one tumor was injected with MBTA therapeutic mixture and it was observed along with non-injected (distal) tumors for their size changes. The growth of MBTA-injected and distal tumors decreased during the first 20 days of therapy, followed by the subsequent growth of both tumors. In line with a significantly reduced tumor growth rate, MBTA-treated mice also demonstrated significantly prolonged survival compared to the control, underscoring MBTA therapeutic efficacy against representative primary and distal tumors. To analyze which immune cells were involved in this disease model and its response to MBTA therapy, we focused on characterizing innate and adaptive immune cell trafficking and infiltration in MBTA-injected and distal tumors, as well as in the spleen. Our data demonstrated that MBTA therapy displays a unique potential to increase innate immune cell infiltration not only in injected but also in distal tumors. MBTA therapy increased the M1/M2 ratio in both the tumors and the spleen. The adaptive immune cell assessment revealed very effective CD4^+^ and CD8^+^ T cell infiltration in both tumor types in MBTA-treated mice, with the highest-level changes observed in the CD8_E/EM_ T cells. Finally, we also demonstrated the importance of CD4^+^ T cells in long-lasting immune memory after MBTA therapy of subcutaneous PHEO. Taken together, these data suggest that the MBTA therapy-induced PHEO growth inhibition could be initiated by a strong innate immune response followed by an adaptive immune response with CD4^+^ T cells preserving long-lasting immune memory after successful treatment.

Systemic immune response during MBTA therapy has been previously observed by Caisova et al. during the therapy of murine metastatic PHEO, where the therapy resulted in slower progression of metastatic lesions and their higher infiltration of CD3^+^ T cells [24]. Medina et al. also described the systemic immune response in a murine bilateral colon carcinoma (CT26) model, in which approximately 30% of the MBTA-treated mice achieved complete tumor regression of both tumors [23]. In this study, similar to murine metastatic PHEO, MBTA therapy did not result in complete tumor elimination, although it contributed to the significant prolongation of the overall survival of treated mice.

These findings led us to focus on the microenvironment in MBTA-injected and distal tumors and analyzed immune cell infiltration during MTBA therapy. The detailed trafficking and infiltration of innate and adaptive immune cells following the treatment showed that MBTA therapy could lead to the conversion of immunologically “cold” to “hot” tumor microenvironments. Taken together, not only were adaptive immune cells elevated in tumors of MBTA-treated mice but also innate immune cells were able to infiltrate the non-injected (distal) tumors. The assessment of innate leukocyte subpopulations indicated higher infiltration of NK cells, monocytes, macrophages, and DCs in both injected and distant tumors. These data are consistent with the previous data from MBTA therapy of the bilateral CT26 model, where DC and monocyte increase also observed in distal tumors [23]. Surprisingly, neutrophils were the only immune cell type elevated solely in the injected tumors. This could be explained by the role of complement receptor 3 and mannan-BAM. After the intratumoral MBTA injection, mannan-BAM anchors into the lipid bilayer of the tumor cells via the hydrophobic properties of BAM, while mannan is recognized by the mannan-binding lectin complement cascade. The resulting activation of the complement cascade induces the proteolytic cleavage of the complement protein C3 into C3a and C3b. Subsequently, the inactive C3b (iC3b) form opsonizes the mannan-BAM-labeled tumor cells. Neutrophils express the receptor CR3, which recognizes iC3b and enables their activation and the frustrated phagocytosis of opsonized tumor cells [30,31]. The lack of neutrophils in non-injected (distal) tumors could also explain the lower response in these tumors during MBTA therapy. However, detailed studies focused on lower response in distal tumors as well as the regrowth of tumors after MBTA therapy are needed.

Classically activated M1-like macrophages exhibit immunostimulatory functions. They are associated with the production of high levels of proinflammatory cytokines and chemokines and the presentation of antigens in the lymphatic nodes. In contrast, alternatively activated M2-like macrophages can reduce inflammation through anti-inflammatory cytokines and promote tumor progression and metastasis [32]. Our data indicate that MBTA therapy triggers macrophage infiltration in the tumors and the spleen while reducing the M2-like phenotype, resulting in a systematically higher M1/M2 macrophage ratio. These findings are consistent with those of other studies where the use of TLR ligands was also associated with a systematically increased M1/M2 ratio [33,34]. In addition, since the anti-CD40 antibody is a component of MBTA therapy, antigen-presenting cells (APCs) that express CD40 receptors, including macrophages, are activated. In the spleen of MBTA-treated mice, we observed a gradual APC increase, namely that of DCs and macrophages, during the entire course of MBTA therapy, suggesting a boosting effect after each cycle of therapy and possibly higher presentation of tumor antigens to the adaptive immune system after each cycle.

It has been established that CD4^+^ T cells also play an important role in developing and sustaining effective antitumor responses, even in cancer immunotherapies primarily designed to activate CD8^+^ T cell responses. The flow cytometry analysis of T cells during MBTA therapy revealed systemic T cell infiltration in the tumors of treated mice, where CD4^+^ T cells became the predominant T cell type. We also show the importance of the CD4^+^ T cells in immune memory after MBTA therapy, using the adoptive transfer of splenic CD4^+^ T cells from previously treated mice. The therapeutic efficacy of CD4^+^ T cells could be attributed to their ability to produce proinflammatory chemo- and cytokines that recruit and activate CD8^+^ T cells and innate immune cells, such as macrophages, to the site of the infection [35,36]. This complex activation of both arms of the immune system could result in a better effector response, for example, after the retransplantation of mice with tumor cells. We have previously shown the role of CD8^+^ T cells in the inhibition of metastatic PHEO lesions during MBTA therapy using T cell depletion antibodies [24]. When CD8^+^ T cells were depleted, metastatic lesion growth in the MBTA-treated group was comparable to that in the control group. These results suggest that CD8^+^ T cells are involved in the eradication of PHEO tumors during MBTA therapy, but CD4^+^ T cells are important for the long-term memory after MBTA therapy of murine PHEO. Similarly, previous results from MBTA therapy of murine pancreatic adenocarcinoma (Panc02) have shown that CD4^+^ T cells are important for the complete eradication of tumor growth and resistance to its recurrence in CD8^−/−^ knockout mice, suggesting that CD4^+^ T cells might play diverse anti-tumor roles in different types of murine tumor models [37]. During the assessment of T cells in bilateral PHEO tumors, only CD8^+^ T cells and their subpopulation of CD8_E/EM_ T cells were elevated in both tumors directly after the first MBTA injection cycle, suggesting a faster acute response in tumors compared to CD4^+^ T cells. Among both the CD4^+^ and CD8^+^ T cells, the effector/effector memory phenotype (CD44^+^) was the most common T cell type not only in the tumors of treated mice but also in the spleen. Previous studies have described the higher proliferation of T cell memory phenotype (CD44^high^) and its antitumor effect during cancer therapies based on a combination of TLR agonists, agonist antibodies, or cytokines [38,39].

B cells play an important role in regulating the immune response to cancer. However, they can produce antibodies against tumor antigens, act as APCs, and produce a variety of immunomodulatory cytokines, thereby activating other cells. Moreover, they can suppress cytolytic responses by acting as regulatory cells and producing immunoregulatory cytokines, such as IL-10 and TGF-β [40]. The exact role of B cells during MBTA therapy is questionable. Previously, we did not observe any B cell increase in tumors during MBT therapy (therapy without anti-CD40) [24]. Since MBTA therapy incorporates anti-CD40 (which polarizes B cells towards an anti-tumor role), it is reasonable to suggest that B cells drive an anti-tumor immune response through their antigen presentation capacity and also the production of anti-tumor antibodies. B cell depletion studies would be certainly required to shed light on this topic, which could be a potential future direction of MBTA immunotherapy.

Understanding the underlying mechanisms that contribute to tumor elimination during MBTA therapy in murine PHEO and other tumor types is indispensable. Here, we present a detailed explanation of immune cell trafficking during intratumoral MBTA therapy to better understand these mechanisms. Despite the potential of this therapy to systematically change the tumor microenvironment into immunologically “hot,” reduce tumor growth, and prolong the survival of treated animals, we could not achieve the complete eradication of murine PHEO tumors with high tumor burden. Our previous study demonstrated that only simultaneous MBTA therapeutic injections into both tumors could potentially treat the bilateral Panc02 model [37]. Therefore, an optimal therapeutic approach, along with MBTA therapy, to increase the antitumor effect in metastatic murine PHEO tumors, is yet to be investigated.

## 5. Conclusions

In conclusion, we showed that intratumoral immunotherapy based on the application of mannan-BAM, TLR ligands, and anti-CD40 antibody (MBTA therapy) exhibits the potential to suppress tumor growth in a murine bilateral pheochromocytoma model. The MBTA therapy could increase the recruitment of innate followed by adaptive immune cells not only into primary (injected) but also into distal (non-injected) tumors. Furthermore, we demonstrated that after successful MBTA therapy of subcutaneous pheochromocytoma, the long-term immunological memory is driven by CD4^+^ T cells. In summary, this study helps to better understand the systemic effect of MBTA therapy and its use for tumor reduction or even elimination, including metastasis.

## Data Availability

The data presented in this study are available on request from the corresponding author.

## Supplementary Materials

The following are available online at https://www.mdpi.com/article/10.3390/cancers13163942/s1, Figure S1: Comparison of immune cell infiltration between injected and non-injected (distal) tumors during MBTA therapy, Figure S2: Representative gating strategy 1, Figure S3: Representative gating strategy 2.
