# Supplementary material for: Identification of Immune Cell Infiltration in Murine Pheochromocytoma during Combined Mannan-BAM, TLR Ligand, and Anti-CD40 Antibody-Based Immunotherapy
Source: Cancers (Basel). 2021 Aug 5;13(16):3942. doi: 10.3390/cancers13163942 (PMC8393500; doi:10.3390/cancers13163942)
Supplement: Supplementary file 1 [file cancers-13-03942-s001.zip › cancers-1302178-supplementary.pdf]

Supplementary materials – Uher et al.

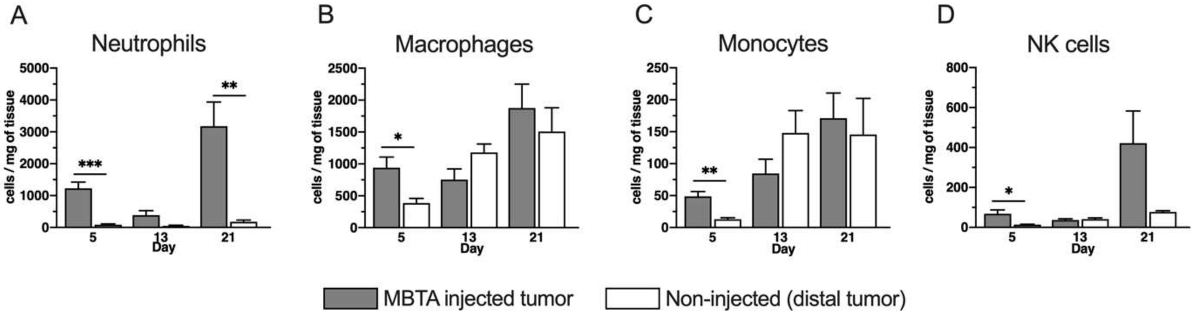

Figure S1. Comparison of immune cells infiltration between injected and non-injected (distal) tumors during MBTA therapy (* $p < 0.05$, ** $p < 0.01$, *** $p < 0.001$).

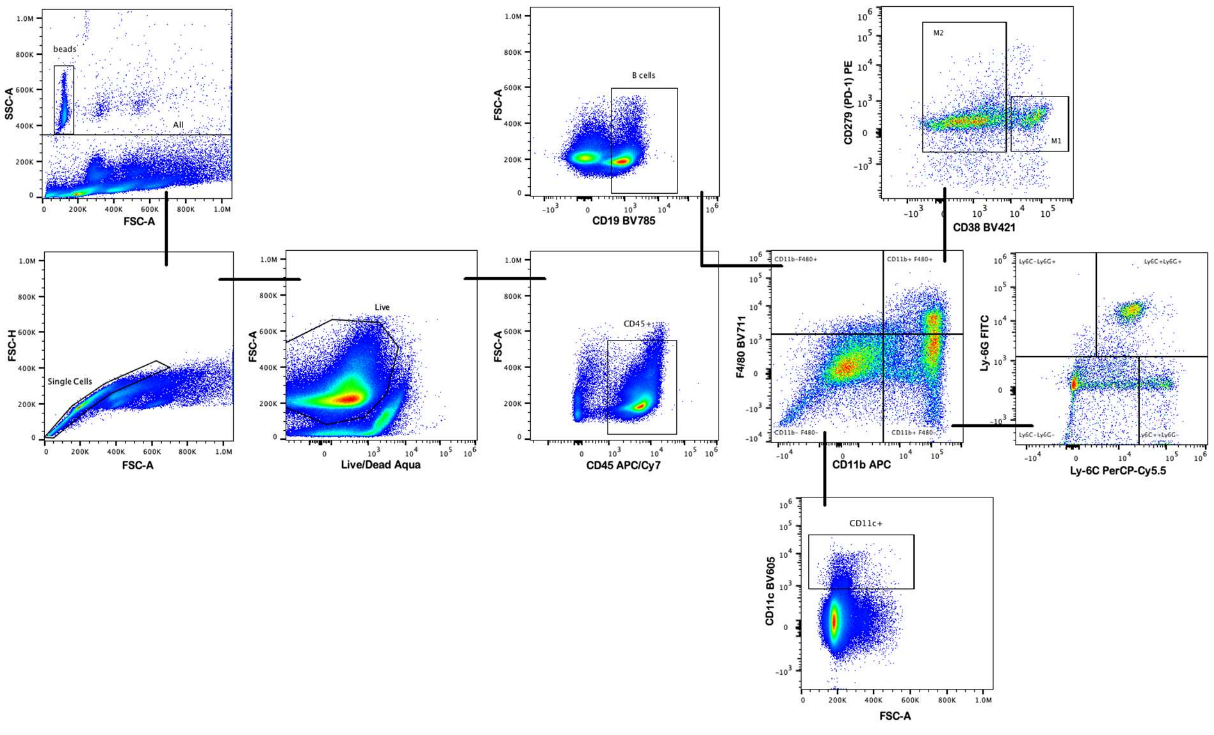

Figure S2. Representative gating strategy 1. Leukocytes ($CD45^{+}$), neutrophils ($CD45^{+}CD11b^{+}F4/80^{-}Ly6G^{+}Ly6C^{lo}$), monocytes ($CD45^{+}CD11b^{+}F4/80^{-}LyG^{-}Ly6C^{hi}$), DCs ($CD45^{+}CD11b^{-}F4/80^{-}CD11c^{+}$), macrophages ($CD45^{+}CD11b^{+}F4/80^{+}$), M1-like macrophages ($CD45^{+}CD11b^{+}F4/80^{+}CD279^{-}CD38^{hi}$), M2-like macrophages ($CD45^{+}CD11b^{+}F4/80^{+}CD279^{+/-}CD38^{lo}$), and B cells ($CD45^{+}CD19^{+}$).

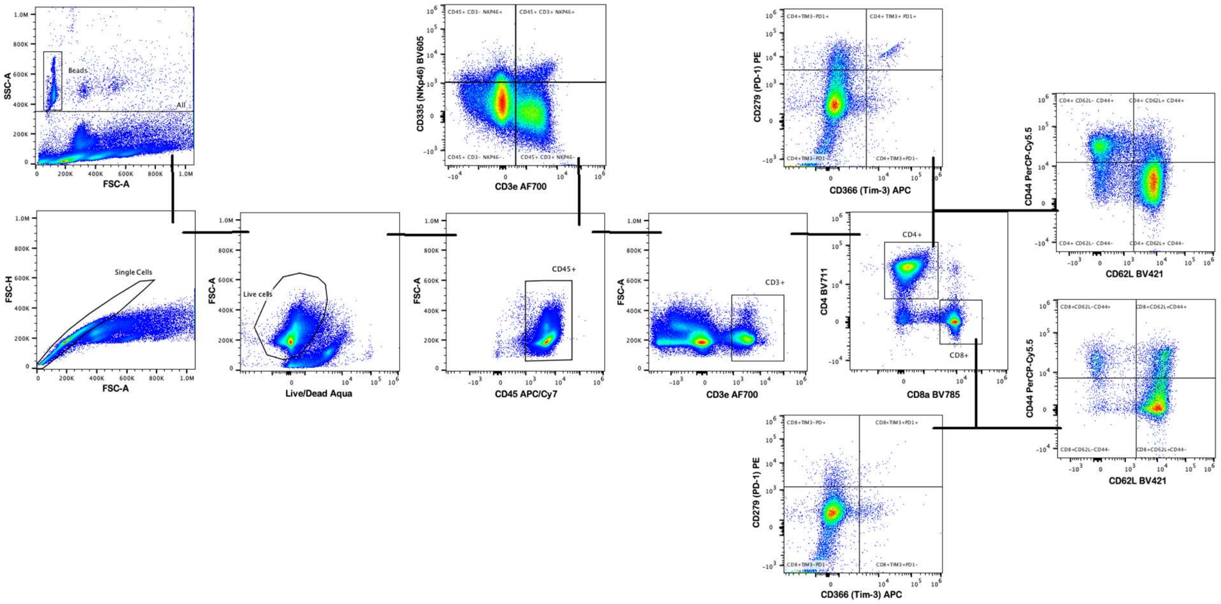

Figure S3. Representative gating strategy 2. CD4^{+} T cells (CD45^{+}CD3^{+}CD4^{+}), CD8^{+} T cells (CD45^{+}CD3^{+}CD8^{+}), CD4$_{E/EM}$ T cells (CD45^{+}CD3^{+}CD4^{+}CD44^{+}CD62L^{-}), CD8$_{E/EM}$ T cells (CD45^{+}CD3^{+}CD8^{+}CD44^{+}CD62L^{-}), CD4$_{CM}$ T cells (CD45^{+}CD3^{+}CD4^{+}CD44^{+}CD62L^{+}), CD8$_{CM}$ T cells (CD45^{+}CD3^{+}CD8^{+}CD44^{+}CD62L^{+}), CD4$_{EX}$ T cells (CD45^{+}CD3^{+}CD4^{+}PD1^{+}TIM3^{+}), CD8$_{EX}$ T cells (CD45^{+}CD3^{+}CD8^{+}PD1^{+}TIM3^{+}), Naïve CD4 T cells (CD45^{+}CD3^{+}CD4^{+}CD44^{-}CD62L^{+}), Naïve CD8 T cells (CD45^{+}CD3^{+}CD8^{+}CD44^{-}CD62L^{+}), and NK cells (CD45^{+}CD3^{+}CD335^{+}).
